# Supplementary material for: Gold price prediction by a CNN-Bi-LSTM model along with automatic parameter tuning
Source: PLoS One. 2024 Mar 7;19(3):e0298426. doi: 10.1371/journal.pone.0298426 (PMC10919698; doi:10.1371/journal.pone.0298426)
Supplement: Attachment — Submitted filename: Response to the comments.docx [file pone.0298426.s002.docx]

**Dear Emily Chenette**

**Editor-in-Chief of PlOS one**

**The authors would like to thank you for your kind reply to our submitted manuscript. We prepared a revised version of our manuscript No. PONEDEC3 upon the useful comments of respected reviewers.**

**Manuscript No. PONEDEC3**

**Response to reviewers' comments**

**The authors would like to thank the reviewer for the careful review of the manuscript, and for the comments, corrections, and suggestions. This document provides a reply to the comments of reviewer. In order to respond, we have added some paragraphs and explained in different sections**

---------------------------------------------------------------------------------------------------------------------

**Reviewer #1:** This study used some existing deep learning models for predicting gold prices.

My comments are as follows.

The main contributions of this study: weak, unsuitable for publication in Plos one. The model verified on a dataset: not convinced yet. The application of this study is limited.

Thank you for your constructive comment. As it is shown in Figure 2, This model has the ability to predict daily gold time series for 44 years with different trends and randomness. This paper shows that the model can effectively predict other commodities prices.

Section 3.1.1. stacked LSTM: I cannot found the stacked? Only LSTM was presented.

Thank you for your constructive comment. The manuscript is revised as recommended (L147). Furthermore, more information about CNN architecture is referenced in section 3.1.2. Other models are constructed of CNN and LSTM models.

Fig 2 and Fig 5 are inconsistent. The yellow part in Fig 2 starts from around 1200. However, Fig 5 starts from 1400.

Thank you for your constructive comment. The difference in the starting value of figures two and five is related to each model's first batch of lag times. As shown in Table 2, all models' lag time is in the range of 10-24 time steps. As a result, each time series's first 10-24 data (based on the optimal lag time of each network) are utilized as lag time and not prediction reference numbers.

**Reviewer #2:**

In this paper, the authors develop a deep learning technique to predict the closing gold prices from all available current observational data. This is an important problem that is working on. It would be reliable to see a comparison between a traditional forecast and state-of-the-art deep learning ways. There are three questions that should be improved as follows:

The training, validation, and test data are divided by period instead of randomly selected. Would this allow any time or financial influence to introduce bias in the trained model? It should be having a discussion here. And there are insufficient statements on the input data, such as the basic indicators.

Thank you for your constructive comment. The manuscript is revised as recommended about the data splitting approach (L 272-277).

The author needs to provide the contributions and the novelty of this study more specific, does this work fill up some knowledge gaps which previous articles cannot address.

Thank you for your constructive comment. The introduction section is revised as recommended (L72-79).

The conclusion and discussion part for this paper are relatively weak, the discussion section is the main part of a paper, but this manuscript mainly reported the other models, in the conclusions, in addition to summarizing the actions taken and results, please strengthen the explanation of their significance.

Thank you for your constructive comment. The conclusion and discussion parts are revised as recommended due to this constructive comment.

**Specific Comments**

Is the manuscript technically sound, and do the data support the conclusions? The manuscript must describe a technically sound piece of scientific research with data that supports the conclusions. Experiments must have been conducted rigorously, with appropriate controls, replication, and sample sizes. The conclusions must be drawn appropriately based on the data presented.

Thank you for your constructive comment. The manuscript (especially the discussion and conclusion) is revised as recommended.

Have the authors made all data underlying the findings in their manuscript fully available?

The PLOS Data policy requires authors to make all data underlying the findings described in their manuscript fully available without restriction, with rare exceptions (please refer to the Data Availability Statement in the manuscript PDF file). The data should be provided as part of the manuscript or its supporting information or deposited to a public repository. For example, in addition to summary statistics, the data points behind means, medians and variance measures should be available. If there are restrictions on publicly sharing data—e.g. participant privacy or use of data from a third party—those must be specified.

Thank you for your constructive comment. Done. The gold price dataset is available at [https://github.com/[amirhosseinse7/Closing-gold-price](https://github.com/amirhosseinse7/Closing-gold-price)](https://github.com/mehriidi/Shemiran_sinoptic_station.git) (L 412).

Is the manuscript presented in an intelligible fashion and written in standard English?

PLOS ONE does not copyedit accepted manuscripts, so the language in submitted articles must be clear, correct, and unambiguous. Any typographical or grammatical errors should be corrected at revision, so please note any specific errors here.

Thank you for your constructive comment. Done. The text has been revised as suggested (changes are available through track change)

With the best regards
