# Supplementary material for: Gold price prediction by a CNN-Bi-LSTM model along with automatic parameter tuning
Source: PLoS One. 2024 Mar 7;19(3):e0298426. doi: 10.1371/journal.pone.0298426 (PMC10919698; doi:10.1371/journal.pone.0298426)
Supplement: Attachment — Submitted filename: Response to reviewers.docx [file pone.0298426.s003.docx]

**Manuscript No. PONEDEC3**

The authors would like to thank you for your kind reply to our submitted manuscript. We prepared a revised version of our manuscript with the title “Gold Price Prediction by a CNN-Bi-LSTM model along with

Automatic Parameter Tuning.” upon the useful comments of respected reviewers.

**Response to reviewers' comments**

---------------------------------------------------------------------------------------------------------------------

At this time, please address the following queries:

a) Please clarify the sources of funding (financial or material support) for your study. List the grants or organizations that supported your study, including funding received from your institution.

b) State what role the funders took in the study. If the funders had no role in your study, please state: “The funders had no role in study design, data collection and analysis, decision to publish, or preparation of the manuscript.”
c) If any authors received a salary from any of your funders, please state which authors and which funders.
d) If you did not receive any funding for this study, please state: “The authors received no specific funding for this work.”
The authors would like to thank the reviewer for the careful review of the manuscript, and for the comments, corrections, and suggestions. The last section is revised as recommended (Funding part) (L415).

Please include your amended statements within your cover letter; we will change the online submission form on your behalf.
4. In your Data Availability statement, you have not specified where the minimal data set underlying the results described in your manuscript can be found. PLOS defines a study's minimal data set as the underlying data used to reach the conclusions drawn in the manuscript and any additional data required to replicate the reported study findings in their entirety. All PLOS journals require that the minimal data set be made fully available. For more information about our data policy, please see http://journals.plos.org/plosone/s/data-availability.

Done. The gold price dataset is available at [https://github.com/[amirhosseinse7/Closing-gold-price](https://github.com/amirhosseinse7/Closing-gold-price)](https://github.com/mehriidi/Shemiran_sinoptic_station.git). It is mentioned at L 416.

"Upon re-submitting your revised manuscript, please upload your study’s minimal underlying data set as either Supporting Information files or to a stable, public repository and include the relevant URLs, DOIs, or accession numbers within your revised cover letter. For a list of acceptable repositories, please see http://journals.plos.org/plosone/s/data-availability#loc-recommended-repositories. Any potentially identifying patient information must be fully anonymized.

Done! We added data as information files and public repository as aforementioned stated.
Important: If there are ethical or legal restrictions to sharing your data publicly, please explain these restrictions in detail. Please see our guidelines for more information on what we consider unacceptable restrictions to publicly sharing data: http://journals.plos.org/plosone/s/data-availability#loc-unacceptable-data-access-restrictions. Note that it is not acceptable for the authors to be the sole named individuals responsible for ensuring data access.
We will update your Data Availability statement to reflect the information you provide in your cover letter.

Additional Editor Comments:
1. The literature review seems poor to me, authors must include recently published articles.
Done! the manuscript has revised as recommended (L130-134) (Two recently published articles have added to this article)

2. Add detail about the features.
The manuscript has revised as recommended (L176-177). About the features which extracted by CNN using time series there are not feature mapping method as there is in image sequence forecasting, so we just add the overall statement about the feature extraction of CNNs.

3. Distinguish your work with the state of the art
The manuscript has revised as recommended (L72-80) and (L135-140).

Reviewers' comments:
Reviewer's Responses to Questions
Comments to the Author
1. Is the manuscript technically sound, and do the data support the conclusions?
The manuscript must describe a technically sound piece of scientific research with data that supports the conclusions. Experiments must have been conducted rigorously, with appropriate controls, replication, and sample sizes. The conclusions must be drawn appropriately based on the data presented.
Reviewer #1: Partly
Reviewer #2: Partly

The manuscript (especially the discussion and conclusion) is revised as recommended. Thank you!
2. Has the statistical analysis been performed appropriately and rigorously?
Reviewer #1: Yes
Reviewer #2: Yes
3. Have the authors made all data underlying the findings in their manuscript fully available?
The PLOS Data policy requires authors to make all data underlying the findings described in their manuscript fully available without restriction, with rare exception (please refer to the Data Availability Statement in the manuscript PDF file). The data should be provided as part of the manuscript or its supporting information, or deposited to a public repository. For example, in addition to summary statistics, the data points behind means, medians and variance measures should be available. If there are restrictions on publicly sharing data—e.g. participant privacy or use of data from a third party—those must be specified.
Reviewer #1: Yes
Reviewer #2: Yes
4. Is the manuscript presented in an intelligible fashion and written in standard English?
PLOS ONE does not copyedit accepted manuscripts, so the language in submitted articles must be clear, correct, and unambiguous. Any typographical or grammatical errors should be corrected at revision, so please note any specific errors here.
Reviewer #1: No
Reviewer #2: Yes

Done. The text has been revised as suggested (changes are available through track change)
5. Review Comments to the Author
Please use the space provided to explain your answers to the questions above. You may also include additional comments for the author, including concerns about dual publication, research ethics, or publication ethics. (Please upload your review as an attachment if it exceeds 20,000 characters)
Reviewer #1: Although the article has corrected some errors in presentation, I keep my opinion as the previous review.
The article's contribution is not convincing enough for publication in PLOS ONE.
I suggest the author to submit the article in other more suitable journals about artificial intelligence.
The article has changed in different sections. I hope this time the article’s contribution is convincing enough for publication in PLOS ONE.
Reviewer #2: Thanks to authors for their contributions.
1. In the introduction: It is clearly stated the gap of research.
2. However, it will be better if they use different data-set source.
Thank you for your suggestion. We utilize different datasets in our future research.

3. In conclusion, authors mentioned certain distinguish features of the proposed model in compare with existing models. ----> I think it is better it should be write more precisely into the result-analysis section using certain statistical information.
Done! The manuscript has been revised as suggested. Thank you!

With the best regards
