# Supplementary material for: Gold price prediction by a CNN-Bi-LSTM model along with automatic parameter tuning
Source: PLoS One. 2024 Mar 7;19(3):e0298426. doi: 10.1371/journal.pone.0298426 (PMC10919698; doi:10.1371/journal.pone.0298426)
Supplement: Attachment — Submitted filename: Response to reviewers.docx [file pone.0298426.s004.docx]

**Dear M. Shamim Kaiser**

**Academic Editor of PlOS one**

**The authors would like to thank you for your kind reply to our submitted manuscript. We prepared a revised version of our manuscript No. PONEDEC3 upon the useful comments of respected reviewers.**

**Manuscript No. PONE-D-22-31551R1**

**Response to reviewers' comments (Revision 2)**

**The authors would like to thank the reviewer for the careful review of the manuscript, and for the comments, corrections, and suggestions. This document provides a reply to the comments of reviewer. In order to respond, we have added some paragraphs and explained in different sections**

---------------------------------------------------------------------------------------------------------------------

**General comment**

It is advised that authors must complete the following:

- The literature review section must be updated.  Done! The recent studies section has been changed as suggested. Thank you for your constructive comment!
- Addressed all the comments of Reviewer 1. The manuscript has been revised as suggested.

**Reviewer #1 comments:**

I still feel that the contribution of the paper is not enough for publication in PLOS ONE.

My suggestions are as follows:

1. Conduct survey of commodity price prediction studies such as:

+ Chen, Yufeng, Jinwang Wu, and Zhongrui Wu. "China’s commercial bank stock price prediction using a novel K-means-LSTM hybrid approach." Expert Systems with Applications 202 (2022): 117370.

+ Liu, Tengteng, et al. "A stock price prediction method based on meta-learning and variational mode decomposition." Knowledge-Based Systems 252 (2022): 109324.

+ Liu, Chong, et al. "An optimized nonlinear grey Bernoulli prediction model and its application in natural gas production." Expert Systems with Applications 194 (2022): 116448.

+ Vo, Anh H., Trang Nguyen, and Tuong Le. "Brent Oil Price Prediction Using Bi-LSTM Network." Intelligent Automation & Soft Computing 26.6 (2020).

+ Pindoriya, N. M., S. N. Singh, and S. K. Singh. "An adaptive wavelet neural network-based energy price forecasting in electricity markets." IEEE Transactions On power systems 23.3 (2008): 1423-1432.

2. Conduct the comparison with state-of-the-art methods for Gold Price Prediction such as:

+ Madziwa, Lawrence, Mallikarjun Pillalamarry, and Snehamoy Chatterjee. "Gold price forecasting using multivariate stochastic model." Resources Policy 76 (2022): 102544.

This research is related to prediction of annual gold price which is less volatile compared to daily gold price. In addition, the researchers used traditional models like AR based model

+ Lu, Wanbo, et al. "International Gold Price Forecast Based on CEEMDAN and Support Vector Regression with Grey Wolf Algorithm." Complexity 2022 (2022).

+ Liang, Yanhui, Yu Lin, and Qin Lu. "Forecasting gold price using a novel hybrid model with ICEEMDAN and LSTM-CNN-CBAM." Expert Systems with Applications 206 (2022): 117847.

Thank you for your constructive comment. Done! The aforementioned studies are added to the manuscript as suggested.

1) Other methods mentioned in part one will be explored in our second article about gold price forecasting using the combination of tuned DNNs, various decomposition algorithms, and ensemble models.

2) Regarding the comparison part, due to the different lengths of test data and overall data, the results of different studies are not quite comparable. For example, in Lu et al. (2022) the researchers just utilized the last 5% of data (in our case last 10%) as test data and the length of the data was different from ours.
