# Supplementary material for: Gold price prediction by a CNN-Bi-LSTM model along with automatic parameter tuning
Source: PLoS One. 2024 Mar 7;19(3):e0298426. doi: 10.1371/journal.pone.0298426 (PMC10919698; doi:10.1371/journal.pone.0298426)
Supplement: Attachment — Submitted filename: Response to reviewers1.docx [file pone.0298426.s005.docx]

**Dear Editor-in-Chief of PlOS ONE**
The authors would like to thank for your kind reply on our submitted manuscript. We prepared revised version of our manuscript (No. PONE-D-22-31551R2) upon the useful comments of respected reviewers. Before listing response to the comment.

**Response to reviewers' comments (Revision 3)**

---------------------------------------------------------------------------------------------------------------------
All comments suggested by the reviewers must be addressed.
The quality of the images is poor. Please improve it.

Done! The quality of all Figures is improved (Manuscript file)

Include recently published articles

Done! the manuscript has revised as (L162-170) (considering Reviewer #2 comments in these studies)

==============================
**Reviewers' comments:**
Comments to the Author
1. If the authors have adequately addressed your comments raised in a previous round of review and you feel that this manuscript is now acceptable for publication, you may indicate that here to bypass the “Comments to the Author” section, enter your conflict-of-interest statement in the “Confidential to Editor” section, and submit your "Accept" recommendation.
Reviewer #1: (No Response)
Reviewer #2: (No Response)
Reviewer #4: (No Response)

2. Is the manuscript technically sound, and do the data support the conclusions?
The manuscript must describe a technically sound piece of scientific research with data that supports the conclusions. Experiments must have been conducted rigorously, with appropriate controls, replication, and sample sizes. The conclusions must be drawn appropriately based on the data presented.
Reviewer #1: Partly
Reviewer #2: Yes
Reviewer #4: Yes

3. Has the statistical analysis been performed appropriately and rigorously?
Reviewer #1: No
Reviewer #2: Yes
Reviewer #4: Yes

4. Have the authors made all data underlying the findings in their manuscript fully available?
The PLOS Data policy requires authors to make all data underlying the findings described in their manuscript fully available without restriction, with rare exception (please refer to the Data Availability Statement in the manuscript PDF file). The data should be provided as part of the manuscript or its supporting information, or deposited to a public repository. For example, in addition to summary statistics, the data points behind means, medians and variance measures should be available. If there are restrictions on publicly sharing data—e.g. participant privacy or use of data from a third party—those must be specified.
Reviewer #1: No
The gold price dataset is available at [https://github.com/[amirhosseinse7/Closing-gold-price](https://github.com/amirhosseinse7/Closing-gold-price)](https://github.com/mehriidi/Shemiran_sinoptic_station.git). It is mentioned in L 416.

Reviewer #2: Yes
Reviewer #4: Yes

5. Is the manuscript presented in an intelligible fashion and written in standard English?
PLOS ONE does not copyedit accepted manuscripts, so the language in submitted articles must be clear, correct, and unambiguous. Any typographical or grammatical errors should be corrected at revision, so please note any specific errors here.
Reviewer #1: No
Done. The text has been revised as suggested.

Reviewer #2: Yes
Reviewer #4: Yes

6. Review Comments to the Author
Please use the space provided to explain your answers to the questions above. You may also include additional comments for the author, including concerns about dual publication, research ethics, or publication ethics. (Please upload your review as an attachment if it exceeds 20,000 characters)
Reviewer #1: I am not satisfied with the author's answer on the comparison part.
Besides, I found the topic of the article to be trivial, nothing new, the scientific quality of the article was not satisfactory for publication in PLOS ONE.
Therefore, I suggest reject.
We have evaluated the Light Gradient Boosted Machine (LightGBM) ensemble model but were unable to improve statistical criteria; therefore, we have omitted this model from our article.

Reviewer #2: Certain observations:
1. Chen et al. [24] ---> you mentioned "more accurate than other methods".
It’s better to mention any metrics that represent the mentioned phrase.
Done. The manuscript has been changed as suggested (L142-144). Thank you!

2. Liu et al. [25] ---> similar explanation like 1.

The text has been modified as suggested (L150)

3. Liang et al. [27] ----> its better to mention the metrics that represent the term "more accurate".
Done. The manuscript has been changed as suggested (L174-175).

The best solution, I think, make a table and mention the result and properties of the mentioned schemes and others.
In order to ensure the comprehensiveness of this table, we think it is better to include other relevant publications that are referenced in the methodology and results sections. This inclusion will necessitate a revision of the overall structure of our article. However, in the event that it is deemed essential, we shall incorporate the aforementioned chart.

Reviewer #4: The paper presents a novel CNN-Bi-LSTM model for closing gold price forecasting. The model is well-designed and the experimental results are convincing. However, there are a few minor concerns that need to be addressed before the paper can be accepted for publication.
1. The paper only uses the closing gold price dataset. It would be interesting to see how the model performs when other influential factors, such as silver price, crude oil, and the U.S. dollar index, are included in the dataset.
As we have previously addressed the multivariate predicting of closing gold price by incorporating several relevant elements in a separate article, we refrain from including these components in the present paper. This topic was addressed in the concluding section. Thank you for this comment!

2. The author could also provide more discussion of the limitations of the model.
The primary constraint of the aforementioned model is its reliance on a substantial volume of data in order to make accurate predictions. This text has been added in L388.

3. The author mentions that data augmentation methods, such as adding noise to the data, may improve the performance of deep neural networks. However, they do not discuss how they might implement these methods in their models or how these augmentation methods might affect the accuracy of the predictions.
The performance of models is contingent upon the quantity of data available and the ability to discern patterns within it. Consequently, augmenting the dataset through the utilization of deep learning models like Generative Adversarial Networks (GANs) or employing data augmentation techniques is expected to enhance the accuracy of the outcomes. Moreover, the application of techniques such as wavelet transform, which involves noise reduction and utilization of the denoised data as input for deep learning models, has the potential to enhance the precision of the outcomes. This description has added in (L459-465).

4. The paper only uses grid search for hyperparameter tuning. It would be beneficial to compare the results of grid search with other hyperparameter tuning methods, such as random search and Bayesian optimization.
The rationale for not comparing alternative hyperparameter tuning approaches in our study was mostly due to the near-perfect performance achieved by the various models under consideration. Additionally, it was anticipated that these alternative methods would converge to the same hyperparameter values as those obtained by grid search.

Overall, the paper is well-written and the research is sound. However, the minor concerns mentioned above need to be addressed before the paper can be accepted for publication.
Thank you!

7. PLOS authors have the option to publish the peer review history of their article (what does this mean?). If published, this will include your full peer review and any attached files.
If you choose “no”, your identity will remain anonymous but your review may still be made public.
Do you want your identity to be public for this peer review? For information about this choice, including consent withdrawal, please see our Privacy Policy.
Reviewer #1: No
Reviewer #2: No
Reviewer #4: No

With the best regards
R. Kalantari
