# Supplementary material for: Gold price prediction by a CNN-Bi-LSTM model along with automatic parameter tuning
Source: PLoS One. 2024 Mar 7;19(3):e0298426. doi: 10.1371/journal.pone.0298426 (PMC10919698; doi:10.1371/journal.pone.0298426)
Supplement: Attachment — Submitted filename: Response to reviewers(1).docx [file pone.0298426.s006.docx]

**Dear Jayesh Soni**

**Academic Editor of PlOS one**

**The authors would like to thank you for your kind reply to our submitted manuscript. We prepared a revised version of our manuscript No. PONE-D-22-31551R3 upon the useful comments of respected reviewers.**

**Manuscript No. PONE-D-22-31551R3**

**Response to reviewers' comments (Revision 4)**

**--------------------------------------------------------------------------------------------------------------------------------------**

Additional Editor Comments:
Your work presents an intriguing approach to predicting gold prices using deep learning techniques. However, I have a few queries that I believe, once addressed, could further strengthen your manuscript:
Given the unpredictability of financial markets, particularly during extraordinary events like the 2008 financial crisis or the recent pandemic, how does your model perform under such atypical conditions? Is there evidence of its adaptability and robustness in these scenarios?
In LSTM-based models, the number of lag times (time steps) determines how and when these models adjust to atypical conditions, such as the ones you described. As Fig. 5 illustrates, different DNN models quickly adjust to the sequence's peaks points (small scale atypical condition) (depending on the lag time, their reaction time changes). However, a detailed analysis of these models' accuracy in atypical scenarios (large scale atypical conditions) will be investigated in future studies.

While your model shows significant promise, a comparative analysis with non-CNN and non-LSTM based machine learning models could offer a broader perspective on its relative performance. Have such comparisons been considered or conducted?
Due to the findings of earlier studies in literature review indicating that DNN-based models outperform ANN models across multiple domains, we do not perform comparative analysis with non-CNN and non-LSTM based machine learning models (such as MLP, RBF, GRNN, etc.) (L59-66)

Looking forward, what are the avenues for enhancement in your model? Are there upcoming methodologies or data sources that could be leveraged to improve prediction accuracy?
Thank you, the text has been changed due to this productive question. The model's performance may be enhanced by incorporating additional variables, such as the price of silver, crude oil, and the US dollar index, or by switching from grid search to other automatic hyperparameter tuning techniques like random search or Bayesian methods. Furthermore, the accuracy of the models may be improved by applying methods like wavelet transform, which reduce noise and use denoised data as input for deep learning models (L456-461).

With the best regards
